# The epidemiology of cruciate ligament rupture in an insured Swedish dog population

**DOI:** 10.1038/s41598-021-88876-3

**Published:** 2021-05-05

**Authors:** Karolina Engdahl, Ulf Emanuelson, Odd Höglund, Annika Bergström, Jeanette Hanson

**Affiliations:** Department of Clinical Sciences, Swedish University of Agricultural Sciences, P.O. Box 7054, 75007 Uppsala, Sweden

**Keywords:** Epidemiology, Ligaments

## Abstract

Cruciate ligament rupture (CLR) is a common orthopedic disorder in dogs. The study objectives were to evaluate incidence rate (IR), cause-specific mortality rate (CSMR) and risk factors for CLR in insured dogs. A single cohort study of dogs insured in Agria Pet Insurance in Sweden (2011–2016) was performed. Age at diagnosis, IR, CSMR and relative risk (RR) for CLR was calculated overall and per breed. The cohort included just over 600,000 dogs. The IR of CLR was 23.8 (95% confidence interval, 23.1–24.6) cases per 10,000 DYAR. The breeds with highest RR of CLR were Boerboel and Dogo Canario, while the breeds with lowest RR were Standard Dachshund and Miniature Pinscher. Dogue de Bordeaux had highest RR of euthanasia due to CLR. The median age at veterinary care claim for CLR was 7.1 (range 0.3–16.0) years and 6.6 (0.3–12) years at life insurance settlement. Large and giant breeds were generally diagnosed and euthanized due to CLR at a younger age compared to smaller breeds. The majority of the breeds with increased RR of CLR diagnosis and CLR-related euthanasia were large or giant. A pattern of increasing size and decreasing age at diagnosis/CLR-related euthanasia was observed.

## Introduction

Cranial cruciate ligament disease (CCLD) is among the most common orthopedic disorders in dogs^1^, and the most common stifle joint disease requiring veterinary care^2^. Many factors, including anatomical configuration, environment and genetics, are suggested to contribute to development of CCLD, and the complex and likely multifactorial origin of the disease makes it challenging to develop preventive strategies^3–5^. The condition can be treated either surgically or conservatively, and there are over 60 variations of surgical procedures described^6^. The treatment is often costly; it was estimated that the total cost of CCLD treatment in the US during 2003 was 1.32 billion dollars^7^. Rupture of the caudal cruciate ligament with intact cranial cruciate ligament is much more uncommon, and is often caused by trauma^1,8^.

The reported prevalence of CCLD varies between 0.56–2.55%^9–11^. However, these prevalence estimates are based on data from veterinary clinics, which may represent a biased proportion of dogs from the general dog population. Population based estimates of disease occurrence are necessary in order to evaluate the impact of CCLD at a population level. Insurance data are feasible for such calculations, given that a sufficient proportion of the dog population is insured. In Sweden, 77% of all dogs were covered by veterinary care insurance during 2012, and during 2016 38% of the dog population was insured in Agria Pet Insurance^12,13^. The Agria Pet Insurance database has been used in epidemiological studies of conditions such as atopic dermatitis, dystocia, epilepsy, and adrenocortical insufficiency^14–17^.

Several breeds such as Rottweiler, Labrador Retriever and Newfoundland are reported as predisposed to CCLD, and the disease often affects middle-aged to older dogs with a reported median age of 4.3–7.0 years at presentation^9,11,18,19^. Females and neutered dogs are generally reported as predisposed to the condition^9–11,19,20^. Comorbidities such as hip dysplasia and patellar luxation have been described in dogs with CCLD, but if these comorbidities act as risk factors for CCLD is not fully evaluated^9,21–23^.

Even though the long-term treatment results for CCLD generally are reported as successful, the disease frequently results in osteoarthritis and chronic pain, which in severe cases could result in euthanasia^24–26^. There is limited information about euthanasia due to CCLD, but one study reported a cause-specific mortality of 2 cases per 10,000 dog-years at risk (DYAR) in dogs insured in Agria Pet Insurance 1995–2000^27^. A study including Norwegian and Swedish dogs treated for CCLD at two University Animal Hospitals reported that 61 of the 333 included dogs (18.3%) were euthanized for reasons related to CCLD at some point after treatment initiation^22^. The most common reason for CCLD-related euthanasia was persistent lameness from the affected limb. Many studies have reported that the risk of CCLD varies with breed, but it is not known if the same applies to the risk of CCLD-related euthanasia.

The objective of the study was to provide population-based estimates of incidence rate, cause-specific mortality rate, and age at diagnosis of cruciate ligament rupture (CLR) for dogs insured in Agria Pet Insurance (Agria Djurförsäkring, Stockholm, Sweden) in Sweden. Additional aims were to investigate whether the affected dogs had claims for other diagnoses prior to the CLR, and if the age at diagnosis and relative risk (RR) of CLR varied with breed. The diagnostic codes of the claims in the Agria Pet Insurance database are based on a hierarchical diagnostic registry developed by the Swedish Association of Veterinary Clinics and Hospitals^28^, and do not differentiate between cranial and caudal cruciate ligament rupture. Caudal cruciate ligament rupture has been reported as much more uncommon than CCLD^1^, and a recent report shows that damage to the caudal cruciate ligament in dogs with CCLD is common, which suggests a mutual underlying pathogenic mechanism^29^. Thus, the results of the current study will be compared to studies evaluating only CCLD.

## Materials and methods

### Data

This was a single cohort study comprising data on dogs insured in Agria Pet Insurance in Sweden between January 1, 2011 and December 31, 2016. There were two types of insurances—veterinary care and life. The data collected included information about breed, sex (female/male, but not neuter status), date of enrollment and termination of the insurance, age at start of the observation period and date and diagnostic codes for veterinary care claims and life insurance settlements (if any). The life insurance terminated at 8, 10 or 12 years of age, depending on breed (see supplementary Table 1). The dogs could be enrolled in veterinary care insurance at any age, but in life insurance only before the age of 4 years (for breeds with insurance termination at 8 years) or 6 years (all other breeds). The age at observation start was based on the median age in the population at 1 January, 2011. The breed variable was based on the classification by Federation Cynologique Internationale (FCI) and the Swedish Kennel Club (SKC). Seven breeds were non-approved by both FCI and the SKC; Old English Bulldog, American Bulldog, Boerboel, Pitbull Terrier, Alaskan Husky, Hedehund and Griffon à Poil Laineux/Boulet. All available breeds were included in the analysis.

The deductible of the veterinary care insurance was chosen by the owner at insurance enrollment, and the claims were registered by a clerk at the company if the total cost of all veterinary appointments during rolling 125-days periods exceeded the deductible. In case receipts from several veterinary appointments were submitted at the same time, they were usually registered as separate claims on the same date. Claims for non-traumatic CLR were reimbursed only after a waiting period of 12 months after insurance enrollment in dogs insured after the age of four months. Two diagnostic codes were included in the database search: “Cruciate ligament rupture” and “Cruciate ligament rupture, several joints”. Since it was not possible to differentiate between cranial and caudal cruciate ligament rupture in the database, different terminology will be used when the results are compared to results from other studies; CLR for the current study, and CCLD for studies evaluating only CCLD.

The age at diagnosis was based on the first registered veterinary care claim for CLR during the observation period or the date when a life insurance settlement was registered in the database. In addition, all claims for other conditions were extracted and included in the analysis for dogs with veterinary care claims for CLR. There was no information about claims before the start of the observation period, and claims for diseases present before insurance enrollment were not reimbursed. Settlement of life insurance required a certificate from the veterinarian, sometimes in combination with an autopsy depending on the cause of death. Euthanasia and natural death were not distinguished in the database. Dogs were excluded in case of uncertain information about age, sex, breed or date of insurance enrollment (for example dogs with date of insurance enrollment before date of birth etc.).

### Analysis

Data analysis was performed in RStudio version 1.2.1335^30^. Continuous variables are presented as median (range) and categorical variables as number (percentage). Wilcoxon rank-sum test was used to compare median age between groups, since the Shapiro–Wilk test showed that the age variable was not normally distributed. Two-sided one sample *z* test of proportions was used to compare proportions. Dog-years at risk (DYAR) was based on the total insurance duration for each dog during the study period (2011–2016). For incidence calculation, the DYAR in dogs with CLR claims during the study period was based on the time period until the first CRL claim. The incidence rates and cause-specific mortality rates were expressed as the number of dogs with veterinary care claims or life insurance settlements due to CLR per 10,000 DYAR. Relative risk for breed was calculated by dividing the incidence rate/cause-specific mortality rate for the breed by the incidence rate/cause-specific mortality rate of the rest of the population (with the breed excluded). Relative risk for sex was calculated similarly. Confidence intervals (CI) were calculated with the R-package “exactci” (version 1.3-3)^31^ based on the Poisson distribution. Bonferroni correction, based on the number of subgroups included in the comparison, was used to adjust for multiple comparisons. P-values < 0.05, after corrections, were considered to indicate statistically significant differences. Breed risks were described using forest plots from the R-package “forestplot” (version 1.9)^32^.

## Results

The database included just over 600,000 insured dogs, and 649 dogs were eliminated due to the exclusion criteria. The majority of the dogs (61.8%) had both veterinary care and life insurance, while the remaining dogs had only veterinary care insurance (35.5%) or life insurance (2.7%). Descriptive features of the dog population are presented in Table 1.

**Table 1:** Descriptive features of dogs insured in Agria Pet Insurance in Sweden, 2011–2016.

	Insurance	
	Veterinary care	Life
Total duration of insurance (years)	> 1.7 million	> 1.1 million
Insurance duration, median (range)*	2.7 y (9.1 w–6.0 y)	2.5 y (9.1 w–6.0 y)
Age at observation start, median (range)	2.4 y (3.4 w–21 y)	1.6 y (3.4 w–12 y)
**Sex (%)**		
Female	49	50
Male	51	50
Number of dogs with claims for CLR	4167	447
CLR	4142	432
Bilateral CLR	45	15
Age at CLR claim, median (range)**	7.1 y (13.3 w–16 y)	6.6 y (16.3 w–12 y)

*Per dog, during 2011–2016.

**Age at first CLR claim during 2011–2016 for dogs with veterinary care insurance.

*CLR* cruciate ligament rupture, *y* years, *w* weeks.

**Figure 1:**
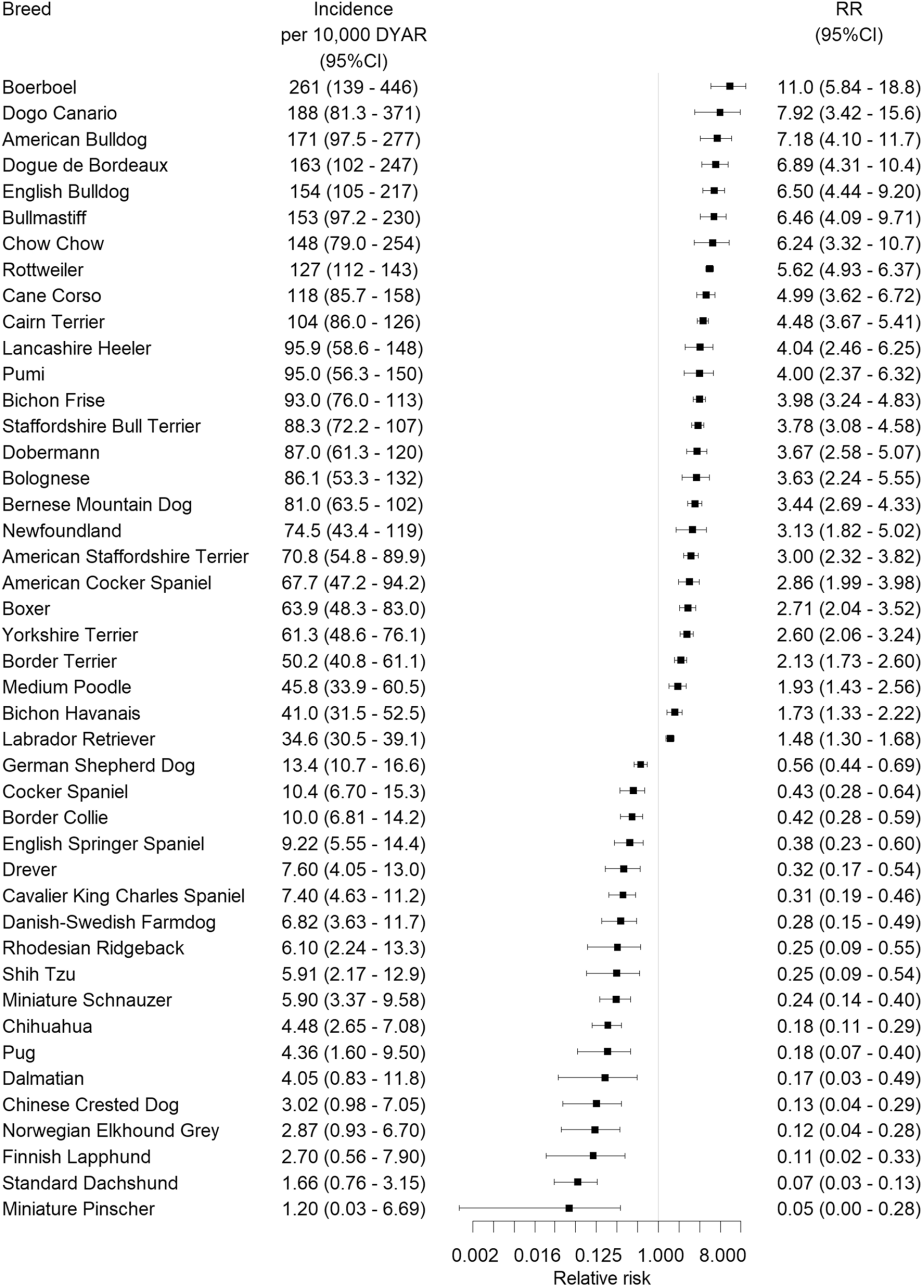
Dog breeds with increased or decreased relative risk (RR) of a veterinary care claim for cruciate ligament rupture (relative to the rest of the population with the breed excluded) in a cohort of dogs insured in Agria Pet Insurance in Sweden (2011–2016). All RRs in the figure were significantly different from 1, after Bonferroni correction based on the number of breeds included in the comparison, n = 339. Note that RR = 0.5 means 2 times decreased risk, RR 0.125 means 8 times decreased risk and so on. *DYAR* dog-years at risk, *CI* confidence interval.

The overall incidence rate of CLR was 23.8 (95% CI, 23.1–24.6) cases per 10,000 DYAR. In total, 181 breeds had at least one dog with a veterinary care claim for CLR. Of these, 26 breeds had significantly increased risk of CLR, while 18 breeds had significantly decreased risk (that is, RR either significantly higher or lower than 1) after Bonferroni correction (Fig. 1, for a full list without Bonferroni correction, see supplementary Table 3). The breeds with highest risk of CLR were Boerboel (RR 11.0, 95% CI, 5.84 – 18.8) and Dogo Canario (RR 7.92, 95% CI, 3.42–15.6) and the breeds with lowest risk were the Standard Dachshund (RR 0.07, 95% CI, 0.03–0.13) and the Miniature Pinscher (RR 0.05, 95% CI, 0.00–0.28).

Female dogs were at increased risk of having a CLR veterinary care claim compared to male dogs (RR 1.13, 95% CI, 1.06–1.20, *p* < 0.001). Of the 4167 dogs with veterinary care claims for CLR, 2762 (66.3%) had a concurrent life insurance. Of these, 466 dogs had a life insurance settlement during the observation period, of which 234 (50.2%) were due to CLR. Median time from CLR veterinary care claim to life insurance settlement due to CLR was 7 days (0–4.25 years). Time from diagnosis of CLR to euthanasia varied with age at diagnosis; it was significantly shorter in dogs diagnosed > 9 years of age, compared to dogs diagnosed < 3 years of age (*p* = 0.0086).

The age at diagnosis and at life insurance settlement due to CLR varied with breed. Breeds with a median age that differed significantly from all other breeds are presented in Table 2. In general, the breeds with significantly lower age at diagnosis were large and giant breeds, while the breeds with significantly higher age at diagnosis were small breeds. The same pattern was seen for the age at euthanasia. The dogs with life insurance settlements due to CLR were significantly younger at time of death/euthanasia, than dogs with life insurance settlement for all other reasons (6.61 (range, 0.31–12.0) years versus 7.53 (0.19–12.83) years, *p* < 0.001).

**Table 2:** Median age in years (range) at first veterinary care claim for cruciate ligament rupture (CLR) or at life insurance settlement due to CLR during the observation period (2011–2016) in a cohort of insured Swedish dogs.

Veterinary care insurance		Life insurance	
Breed	Age* at first CLR claim	Breed	Age at CLR claim
Boerboel	1.98 (1.08–3.98)	Dogo Canario	1.86 (1.81–1.92)
English Bulldog	2.65 (0.79–9.63)	English Bulldog	2.63 (1.93–4.11)
French Bulldog	2.67 (0.25–10.3)	Dogue de Bordeaux**	3.11 (1.48–8.42)
Cane Corso	2.68 (0.56–9.11)	Cane Corso	3.15 (1.08–8.71)
American Staffordshire Terrier	3.56 (0.44–11.5)	Bullmastiff	3.43 (1.30–4.93)
American Bulldog	3.56 (1.44–7.79)	Newfoundland	3.97 (1.25–6.81)
Bullmastiff	3.72 (0.95–6.95)	Rottweiler	4.99 (0.65–9.87)
Dogue de Bordeaux	3.92 (1.01–7.06)	Medium Poodle	8.98 (7.87–11.5)
Staffordshire Bull Terrier	4.75 (0.50–13.6)	Bolognese	9.07 (7.24–9.93)
Boxer	4.92 (0.29–10.4)	Cairn Terrier	9.22 (7.08–10.6)
Rottweiler	5.15 (0.30–11.6)	Miniature and Toy Poodle	9.30 (1.62–10.0)
Jack Russell Terrier	8.44 (0.78–14.5)	Standard Poodle	9.90 (9.72–10.1)
Bichon Frise	8.81 (2.90–14.5)	Soft Coated Wheaten Terrier	10.5 (9.11–12.0)
Border Terrier	9.37 (1.61–13.1)	Border Terrier	10.9 (9.41–11.9)
Miniature and Toy Poodle	9.38 (0.73–13.8)	West Highland White Terrier	11.6 (10.3–11.8)
Cairn Terrier	9.41 (2.18–15.3)		
Pumi	9.48 (7.05–12.2)		
Medium Poodle	9.59 (2.20–13.9)		
West Highland White Terrier	11.1 (5.13–13.2)		
Tibetan Spaniel	11.3 (2.68–12.7)		

*The median age at veterinary care claim for CLR in the listed breeds differed significantly from the median age in all other breeds after Bonferroni correction (based on the number of breeds included in the comparison, n = 181). For a full list of breeds with significantly higher/lower median age at veterinary care claim without Bonferroni correction, see supplementary Table 2.

**The only breed with median age at CLR life insurance settlement that differed significantly from the median age of all other breeds, after Bonferroni correction (based on the number of breeds included in the comparison, n = 99). The other listed breeds had a median age at life claim that differed significantly from the median of all other breeds, without Bonferroni correction.

In total, 2656 of the 4167 (63.7%) dogs with veterinary care claims for CLR had previous claims for other diseases. The frequency and distribution of veterinary care claims by organ system is presented in Table 3, with comparison to claims in veterinary care-insured dogs without CLR. Of the 4167 dogs with CLR, 1542 (37.0%) had previous claims for musculoskeletal disorders and 1093 (26.2%) for dermatologic conditions. The most common diagnoses within these organ systems were joint disease and neoplastic skin disease, which affected 913 (21.9%) and 292 (7.01%) of the dogs with veterinary care claims for CLR, respectively (Tables 4 and 5).

**Table 3:** Previous diagnoses by organ system in dogs with and without cruciate ligament rupture (CLR), in a cohort of dogs insured in Agria Pet Insurance (2011–2016).

Organ system	% of dogs with CLR**	% of dogs without CLR***
Musculoskeletal*	37.0	10.3
Dermatologic*	26.2	17.7
Gastrointestinal*	17.1	15.3
Other (general/unspecific)*	13.5	11.2
Urogenital*	11.8	10.3
Opthalmic*	5.40	4.19
Respiratory	3.53	3.27
Neurologic	2.16	2.02
Hepatic*	2.06	1.43
Endocrine	1.54	1.08
Cardiovascular	1.51	1.62
Hematopoietic	1.10	1.31

*The proportion of dogs with diagnoses in these organ systems was significantly higher in dogs with CLR, after Bonferroni correction based on the number of organ systems, n = 12.

**In total 4167 dogs with veterinary care claims for CLR.

***All dogs with veterinary care insurance, except for the dogs with CLR claims.

**Table 4:** Previous musculoskeletal diagnoses in dogs with veterinary care claims for cruciate ligament rupture (CLR) in Agria Pet insurance (2011–2016).

Category	% of dogs with CLR*
Joint disease	21.9
*Stifle*	15.6
Pain/signs without confirmed cause	8.18
Degenerative changes	3.14
Arthritis	2.30
Patellar luxation	2.14
Meniscal injury	0.79
Traumatic injuries	0.70
Osteochondrosis	0.22
Fracture	0.02
Neoplastic disease	0.02
*Hip*	*2.93*
Hip dysplasia	0.84
*Elbow*	2.26
Degenerative changes	0.98
*Phalanges*	1.30
Degenerative changes	0.26
*Several (unspecified) joints*	1.13
Degenerative changes	0.34
*Shoulder*	*0.79*
Osteochondrosis	0.17
*Carpus*	0.74
Traumatic injuries	0.24
*Tarsus*	0.55
Traumatic injuries	0.12
Lameness/stiffness (unspecified)	18.1
Back/vertebrae	3.84
Spondylosis	0.82
Other	2.40
Fracture/fissure	0.89
Muscle/tendon/bursa	1.44
Traumatic injuries	0.41

*In total 4167 dogs with veterinary care claims for CLR.

All subcategories for stifle joint diagnoses are presented. For all other categories the most specific subcategory is presented, even though pain/signs without confirmed cause was generally the most common subcategory.

**Table 5:** Previous dermatologic diagnoses in dogs with veterinary care claims for cruciate ligament rupture (CLR) in Agria Pet insurance (2011–2016).

Category	% of dogs with CLR*
Neoplastic disease	7.01
Dermatitis	6.77
Ear disease	6.29
Traumatic injury	5.47
Claw disease	3.79
Allergic disease	2.78
Pruritus	2.76
Other	1.82
Parasitic infection	0.55
Alopecia	0.34
Seborrhoea	0.10
Autoimmune disease	0.07

*In total 4167 dogs with veterinary care claims for CLR.

**Figure 2:**
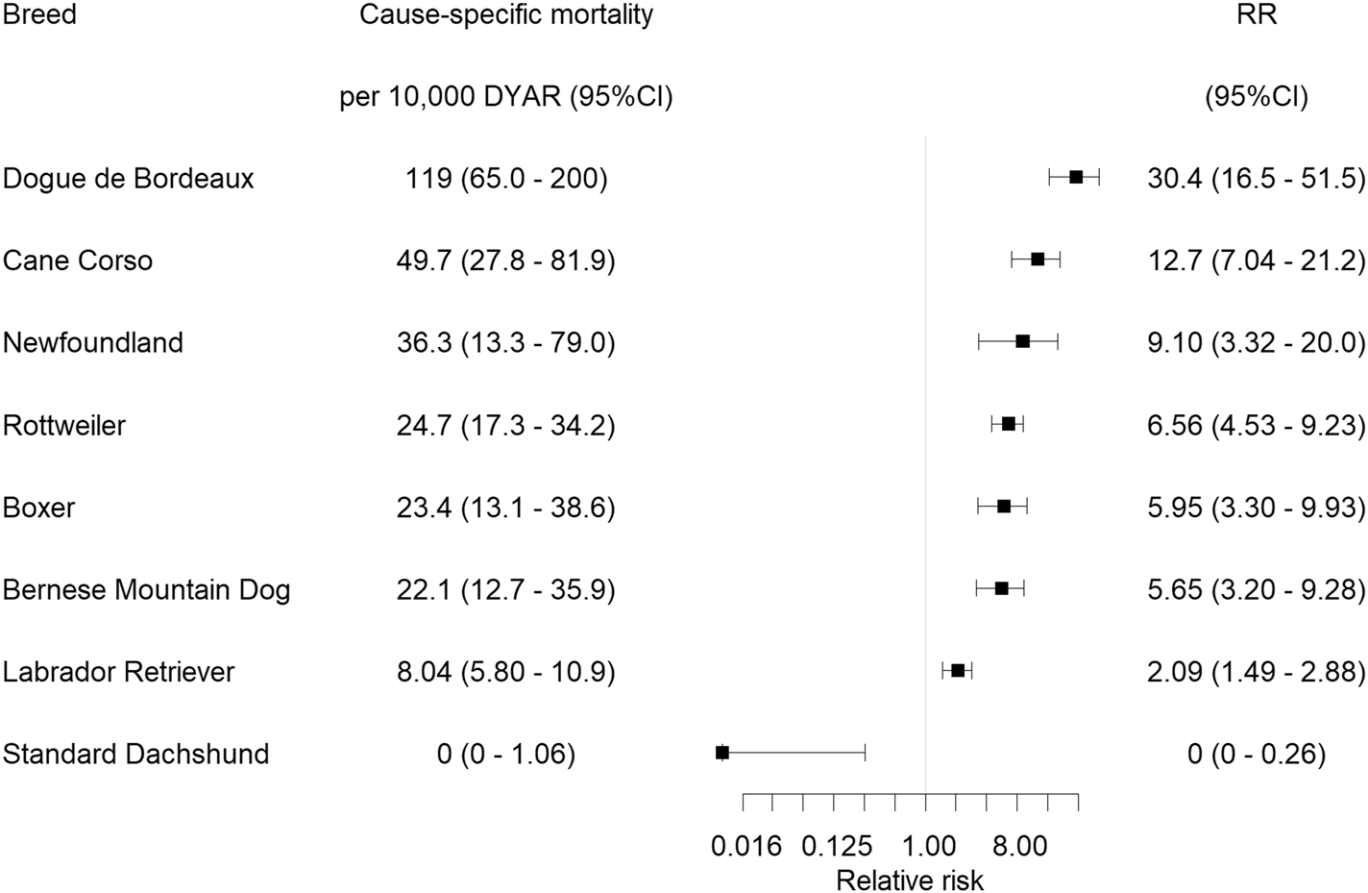
The breeds with increased or decreased relative risk (RR) of death/euthanasia due to cruciate ligament rupture (relative to the rest of the population with the breed excluded) in a cohort of dogs insured in Agria Pet Insurance in Sweden during 2011–2016. All RRs in the figure were significantly different from 1, after Bonferroni correction based on the number of breeds included in the comparison, n = 335. Note that RR = 0.5 means 2 times decreased risk, RR 0.125 means 8 times decreased risk and so on. A fudge factor of 0.01 was added to the RR of Standard Dachshund in order to present the RR on the log-scaled x axis. *DYAR* dog-years at risk, *CI* confidence interval.

The cause-specific mortality rate was 4.04 (95% CI, 3.67–4.43) deaths per 10,000 DYAR, and 99 breeds had at least one dog with a life insurance settlement due to CLR. There were 7 breeds with significantly increased RR and 1 breed with significantly decreased RR of euthanasia due to CLR after Bonferroni correction (Fig. 2 and supplementary Table 4, which consist a full list without Bonferroni correction). The breeds with highest risk of euthanasia due to CLR were the Dogue de Bordeaux (RR 30.4, 95% CI, 16.5–51.5) and the Cane Corso (RR 12.7, 95% CI, 7.04–21.2), while the breed with lowest risk was the Standard Dachshund (RR 0, 95% CI, 0–0.26). There was no difference in risk of euthanasia due to CLR in females compared to males (RR 1.12, 95% CI, 0.93–1.36, *p* = 0.24). Approximately half of the dogs with life insurance settlements due to CLR (52.3%) had a veterinary care claim for CLR at some point before the life insurance settlement.

## Discussion

The current study demonstrated large breed-specific differences in incidence rate, cause-specific mortality rate and age at diagnosis of CLR. The breeds with highest RR of CLR were Boerboel, Dogo Canario, American and English Bulldog, Dogue de Bordeaux, Bullmastiff, Chow Chow, Rottweiler and Cane Corso. Some of these breeds, such as the Rottweiler, English Bulldog and Chow Chow, are commonly reported as predisposed to CCLD in other studies, together with Newfoundland, Boxer, Staffordshire Bull Terrier, American Staffordshire Terrier and Yorkshire Terrier, which all are among the high-risk breeds in the current study^9,11,19^. Several of the identified low-risk breeds, for example the Cocker Spaniel, German Shepherd Dog, Chihuahua, Miniature Schnauzer, Shih Tzu, English Springer Spaniel, Pug and Standard Dachshund, are also reported as low-risk breeds in other studies^11,19,20^. Previous reports about the risk of CCLD for the Labrador Retriever are conflicting; some studies report the Labrador Retriever as a high-risk breed^9,19,20^, while others found no increased risk^11,33^. In the current study, the Labrador Retriever had a slightly increased RR of CLR. The difference in results may be due to differences in study design and study populations, or regional differences in disease pattern.

Some of the high-risk breeds in the current study, such as Boerboel and Dogo Canario, have not been previously reported as predisposed to CCLD. These breeds were represented by relatively few individuals in the insured dog population, but increased in popularity during the observation period (based on increasing DYAR/year, data not shown). It is possible that these breeds were too uncommon to be identified as high-risk breeds in older studies, and that more recent studies, but with smaller study populations, had too low power to identify these breeds as high-risk. Our findings may also be a result of regional differences in disease pattern, or an actual increase in CLR incidence within these breeds, but our data was not sufficient to investigate such time trends.

The majority of the breeds with highest RR of CLR were large or giant breeds, even though several small breeds such as Bichon Frise, Bolognese and Yorkshire Terrier were among the high-risk breeds. The breeds with low risk of CLR also varied in size from small to large, but the giant breeds were only represented among the high-risk breeds. This indicates that other factors than body size contribute to the development of CCLD, but that giant dogs may be at higher risk compared to dogs of smaller sizes. The fact that certain breeds have increased risk of CLR, irrespective of size, suggests a genetic component to the etiology of the disease, which already has been shown for CCLD in the Newfoundland and the Labrador Retriever^4,34^. The same pattern was seen among the dogs with increased risk of euthanasia due to CLR, where all of the high-risk breeds were large or giant. Increasing body weight has been associated with higher risk of euthanasia due to CCLD^22^. This association may be explained by several factors. For example, the risk for postoperative complications after surgical treatment of CCLD increase with increasing body weight^35–41^, and such complications could potentially result in euthanasia in severe cases. In addition, the recovery period after CCLD in a large, heavy dog may be challenging for the owners, as the dog may need to be carried up stairs etc., which can be difficult given the dog’s weight. This could also contribute to a decision of euthanasia.

Female dogs were at increased risk of CLR compared to male dogs in the current study, which is supported by previous reports^10,19^. However, many studies report increased risk of CCLD in neutered dogs compared to entire^9,11,20^. The effect of neutering could not be evaluated in the current study, due to lack of information about neuter status in the insurance database. In a survey of the Swedish dog population performed > 20 years ago, only 7.2% of the female dogs and 3.7% of the male dogs were neutered^42^. A more recent study conducted during the study period (2012) concluded that 22.3% (± 4.8%) of the dogs were neutered, with no separation of female/male dogs^12^. Neuter status could potentially have a confounding effect on the association between sex and CLR, if females were neutered to a higher extent than males in the study population.

The median age at diagnosis of CLR was 7.1 years, which is largely in accordance with previous studies^11,18^. There were large breed-specific differences in age at diagnosis; the majority of the breeds with significantly lower age at diagnosis were large or giant, while the majority of the breeds with significantly higher age at diagnosis were small. Information about age at CLR diagnosis by breed is limited in the literature, but a pattern of increasing weight and decreasing age at diagnosis has been described^19,20^. Rottweilers have been reported as younger than other breeds at time of presentation^18^. In addition, several of the breeds with a significantly lower age at diagnosis in the current study (English Bulldog, American Staffordshire Terrier, and Rottweiler) were reported as predisposed to CCLD in a study investigating breed as a risk factor for the CCLD in dogs under two years of age^20^. The age at euthanasia due to CLR showed a similar pattern of increasing size of the breed and decreasing age at euthanasia. Larger dogs generally have a shorter lifespan than smaller, since increasing body weight is negatively correlated with longevity^43^. Thus, a large breed dog may be perceived as “old” at a younger age compared to a smaller dog, which may affect the treatment recommendation by the examining veterinarian and the owner’s decision of euthanasia. However, this was probably not the main reason for euthanasia, since the median age at time of euthanasia in several of the large and giant breeds was below four years. Further studies on the connection between age, breed and CLR are warranted.

About 2/3 of the dogs with veterinary care insurance claims for CLR had previous claims for other diseases. Almost 16% had claims for stifle joint disease, and the most common diagnoses were related to pain/signs without confirmed cause, degenerative joint disease and arthritis. It is likely that some of these diagnoses were in fact undiagnosed CCLD, as the cranial drawer test used to diagnose CCLD can be negative in a conscious dog despite a ruptured ligament^44^. Another explanation is that the CCLD is the result of a chronic degenerative process, which may have been a reason for the preceding visits^29,45^. Some diagnoses, i.e. within the musculoskeletal, dermatologic, gastrointestinal, urogenital, ophthalmic and hepatic organ system as well as other, general diagnoses affecting the whole body (such as fatigue), were more common in dogs with CLR compared to dogs without CLR. It is possible that some of these disorders or their associated treatments predisposed to CLR. For example, glucocorticoids are commonly prescribed for long-term treatment of conditions such as atopic dermatitis, and it is known that ligament rupture is a clinical manifestation of hyperadrenocorticism in dogs^46–48^. It is also possible that these diagnoses share common risk factors with CLR. For example, large or giant breeds are reported as more likely to develop both HD, osteoarthritis and CCLD^9,49^. Causal inference between previous diagnoses and the subsequent CLR cannot be drawn, due to the retrospective nature of the study. Despite this, it is important to know that many dogs with CLR had previous or concurrent comorbidities at time of diagnosis. These comorbidities could affect the prognosis for returning to adequate function and mobility, and thus affect the recommendation/decision of treatment by the examining veterinarian and the owner.

There is limited information about cause-specific mortality of CLR in the literature. Even though one should be cautious with direct comparisons between studies, the cause-specific mortality rate of 4.04 (95% CI, 3.67–4.43) deaths per 10,000 DYAR in the current study was higher than the 2 deaths per 10,000 DYAR reported in a study of mortality in the Agria Pet Insurance database 1995–2000^27^. One possible explanation for the increased mortality is increasing veterinary tariffs and the development of advanced surgical treatment options for CCLD. This causes an increased financial burden on the animal owner, who may have chosen euthanasia over a high cost treatment to a higher extent during the study period than > 20 years ago. Of the dogs with CLR life insurance settlements, only 53.1% had a previous veterinary care claim for CLR. It is possible that the dogs without a previous veterinary care claim for CLR had gone through a nonsurgical treatment of the CLR with costs not reaching the deductible of the insurance, since nonsurgical treatment of CLR generally is less costly than surgical treatment^7^. Another possible explanation is that some of the dogs had a CLR veterinary care claim before the start of the observation period. Still, it is likely that some dogs were euthanized at time of CLR diagnosis. Over 50% of the dogs that had a veterinary care claim for CLR and a subsequent life insurance settlement were euthanized due to CLR. The median time from CLR diagnosis to CLR-related euthanasia was 7 days. Some dogs were euthanized at the day of CLR diagnosis, while the maximum time between diagnosis and euthanasia was 4.25 years. The fact that some dogs had several weeks or years between diagnosis and euthanasia may imply that treatment failures occur, and result in euthanasia.

Although insurance data are valuable for epidemiology research, some limitations should be mentioned. Agria Pet Insurance database was validated against practice records > 20 years ago^50^. The validation showed excellent agreement for sex and breed but fair agreement for birth date, with a tendency of better agreement for clinics with computerized medical records. Since computerized medical records are used in the majority of Swedish clinics today, the current agreement is most likely better. The dataset generally has high statistical power, but significant associations in breeds represented by few individuals will have been found only if the CCLD incidence of the breed is very high. Even though the dataset is large, it is important to consider the precision of the estimates when the results are interpreted, which can be done by evaluating the width of the confidence intervals.

The reporting of claims relies on the examining veterinarians, who all have different routines for clinical examinations and diagnostic procedures. There is a risk that some of the dogs with CLR were reported under more unspecific diagnostic codes, such as “Lameness, without further specification”. Thus, the incidence rate and cause-specific mortality rate of CLR are probably slightly underestimated. There is also a risk that age at life insurance settlement is underestimated, if dogs were euthanized due to CLR after termination of the life insurance. Increasing bodyweight within breed has been associated with increased odds of CCLD^11^, but could not be evaluated in the current study due to lack of information in the database.

Even though there was a separate diagnostic code for bilateral cruciate ligament rupture, it was rarely used. One study of Labrador Retrievers with CCLD reported a frequency of bilateral rupture at initial presentation of 10.6%, and that subsequent rupture occurred in almost 50% of the dogs^51^. In another study, 54% of the dogs developed contralateral CCLD^52^. It is likely that the code “Cruciate ligament rupture” was used for dogs with bilateral rupture in the current study, especially in case of subsequent rupture. Consequently, the true occurrence of bilateral CLR was probably higher than reported. Contralateral CLR has been shown to affect the decision of euthanasia in dogs with CCLD^22^. Thus, there is a risk that bilateral rupture had a confounding effect on the association between breed and mortality due to CLR, if some breeds were affected by bilateral rupture to a higher extent than others.

Morbidity and mortality of insured dogs may not reflect mortality and morbidity in uninsured animals^53^. For example, a study that evaluated CCLD in dogs attending primary-care practices in England reported increased odds of a diagnosis in insured dogs, compared to in uninsured^11^. It is not known if the same pattern exists in dogs insured in Sweden. However, the results of our study are probably representative for the majority of the Swedish dog population due to the high insurance coverage, although a selection bias in the choice of insurance company may exist.

## Conclusion

In conclusion, CLR affected more than 4000 dogs in the population, with an incidence of 23.8 (95% CI, 23.1–24.6) cases per 10,000 DYAR. Although dogs of all sizes were affected, the majority of the breeds with increased RR of CLR were large or giant. Large and giant breeds also had an increased risk of euthanasia due to CLR, and were generally diagnosed and euthanized due to CLR at a younger age compared to smaller breeds. Demographic factors associated with CLR provide guidance for veterinarians in their clinical work, and may educate breeders and dog owners about breeds at risk of disease. In addition, the results may guide studies investigating the aetiopathogenesis of CLR. Given the identified breed predispositions, which likely has a genetic component, breeds reforms may be warranted in the future to lower the incidence of CLR.

## Supplementary Information

Supplementary Information.

## Data Availability

The data analyzed in the current study are not publicly available due to a non-disclosure agreement with Agria Pet Insurance.
