# Supplementary material for: The epidemiology of cruciate ligament rupture in an insured Swedish dog population
Source: Sci Rep. 2021 May 5;11:9546. doi: 10.1038/s41598-021-88876-3 (PMC8100293; doi:10.1038/s41598-021-88876-3)
Supplement: Supplementary file 1 — Supplementary Information. [file 41598_2021_88876_MOESM1_ESM.pdf]

The epidemiology of cruciate ligament rupture in an insured Swedish dog population

Karolina Engdahl[1]* DVM; Ulf Emanuelson[1], MSc, PhD; Odd Höglund[1], DVM, PhD; Annika Bergström[1] DVM, DECVS, PhD; Jeanette Hanson[1] DVM, DECVIM, PhD

[1]Department of Clinical Sciences, Swedish University of Agricultural Sciences, P.O. Box 7054, 75007 Uppsala, Sweden

*Corresponding author: Karolina Engdahl, karolina.engdahl@slu.se

Supplementary table 1. Age at termination of life insurance by breed, for dogs insured in Agria Pet Insurance (2011-2016)

Age group 12	Bichon Havanais, Border Terrier, Cairn Terrier, Chihuahua, Chinese Crested, Miniature Schnauzer, Finnish Lapphund, Finnish Spitz, Fox Terrier, Islandic Sheepdog, Jack Russel Terrier, Lhasa Apso, Poodle (toy, miniature, medium), Münsterländer, Norrbottenspets, Norwegian Buhund, Papillon, Phalene, Schnauzer, Shih Tzu, Soft Coated Wheaten Terrier, Tibetan Spaniel, Tibetan Terrier, Västgötaspets, Welsh Springer Spaniel, West Highland White Terrier, Whippet
Age group 10	All other breeds
Age group 8	Berner Sennen, Grand Danois, Irish Wolfhound, Leonberger, Newfoundland, Pyrenean Mountain Dog, Neapolitan Mastiff, St. Bernard

Supplementary table 2. Median age at first CLR veterinary care claim during the observation period (2011-2016) in a cohort of insured Swedish dogs, for breeds with median age that differed significantly from the median age of all other breeds ($p < 0.05$).

Breed	Median age
Salukis	1.45
Boerboel*	1.98
Bull Terrier	2.42
Dogo Canario	2.43
Leonberger	2.52
English Bulldog*	2.65
French Bulldog*	2.67
Cane Corso*	2.68
St. Bernhards	3.39
American Bulldog*	3.56
American Staffordshire Terrier	3.56
Bullmastiff*	3.72
Dogue de Bordeaux*	3.92
Chow Chow	3.97
English Mastiff	4.22
Newfoundland	4.61
Hovawart	4.68
Staffordshire Bull Terrier*	4.75
Boxer*	4.92
Great Dane	5.15
Rottweiler*	5.15
Dobermann	5.26
Swedish Elkhound	5.74
German Shepherd Dog	6.11
American Cocker Spaniel	7.79
Yorkshire Terrier	7.88
Bichon Havanais	8.00
Jack Russell Terrier*	8.44
Bichon Frise*	8.81
Cavalier King Charles Spaniel	8.89
Fox Terriers	9.00
Beagle	9.11
Coton de Tuléar	9.14
Border Terrier*	9.37
Poodle Miniature & Toy*	9.38
Cairn Terrier*	9.41
Pumi*	9.48
Poodle Medium*	9.59
Norwich Terrier	9.72
Stabyhound	9.76
Japanese Spitz	9.82
Danish-Swedish Farmdog	10.2
Papillon	10.3
Dachshunds Standard	10.3
Shiba Inu	10.4
Münsterländer Small	11.0

West Highland White Terrier*	11.1
Tibetan Spaniel*	11.3
Parson Russell Terrier	11.9
Schipperke	12.2

*significanly older/younger after Bonferroni correction

Supplementary table 3. Breeds with increased or decreased relative risk of a veterinary care claim for cruciate ligament rupture (relative to the rest of the population with the breed excluded) in a cohort of dogs insured in Agria Pet Insurance in Sweden during 2011-2016. All RRs were significantly different from 1 ($p < 0.05$).

Breed	Relative risk (95% CI)
Fila Brasileiro	26.9 (5.55 - 78.7)
Berger de Picardie	19.9 (4.11 - 58.3)
Neapolitan Mastiff	15.0 (1.82 - 54.2)
Perro de Presa Mallorquin/ca de Bo	13.0 (1.57 - 46.9)
Boerboel*	11.0 (5.84 - 18.8)
Entlebucher Sennenhund	8.71 (1.79 - 25.5)
Dogo Canario*	7.92 (3.42 - 15.6)
American Bulldog*	7.18 (4.10 - 11.7)
Dogue de Bordeaux*	6.89 (4.31 - 10.4)
Caucasian Shepherd Dog	6.55 (1.78 - 16.8)
English Bulldog*	6.50 (4.44 - 9.20)
Bullmastiff*	6.46 (4.09 - 9.71)
English Mastiff	6.33 (2.05 - 14.8)
Chow Chow*	6.24 (3.32 - 10.7)
Rottweiler*	5.62 (4.93 - 6.37)
Cane Corso*	4.99 (3.62 - 6.72)
Cairn Terrier*	4.48 (3.67 - 5.41)
American Akita	4.05 (2.02 - 7.25)
Lancashire Heeler*	4.04 (2.46 - 6.25)
Pumi*	4.00 (2.37 - 6.32)
Bichon Frise*	3.98 (3.24 - 4.83)
Staffordshire Bull Terrier*	3.78 (3.08 - 4.58)
Dobermann*	3.67 (2.58 - 5.07)
Bolognese*	3.63 (2.24 - 5.55)
Bernese Mountain Dog*	3.44 (2.69 - 4.33)
Newfoundland*	3.13 (1.82 - 5.02)
American Staffordshire Terrier*	3.00 (2.32 - 3.82)
American Cocker Spaniel*	2.86 (1.99 - 3.98)
Boxer*	2.71 (2.04 - 3.52)
Yorkshire Terrier*	2.60 (2.06 - 3.24)
Swedish Lapphund	2.47 (1.13 - 4.69)
Great Pyrenees	2.41 (1.10 - 4.57)
Border Terrier*	2.13 (1.73 - 2.60)
Poodle Medium*	1.93 (1.43 - 2.56)
Bichon Havanais*	1.73 (1.33 - 2.22)
Beagle	1.60 (1.08 - 2.27)
Labrador Retriever*	1.48 (1.30 - 1.68)
Jack Russell Terrier	1.31 (1.09 - 1.55)
Swedish Elkhound	0.63 (0.48 - 0.81)
Soft Coated Wheaten Terrier	0.56 (0.33 - 0.89)
Wachtelhund	0.56 (0.35 - 0.86)
German Shepherd Dog*	0.56 (0.44 - 0.69)
Lagotto Romagnolo	0.54 (0.26 - 0.99)
Shetland Sheepdog	0.54 (0.35 - 0.79)
Papillon	0.51 (0.29 - 0.83)

Whippet	0.51 (0.25 - 0.91)
Parson Russell Terrier	0.47 (0.20 - 0.93)
Poodle Standard	0.45 (0.26 - 0.73)
Cocker Spaniel*	0.43 (0.28 - 0.64)
Petit Basset Griffon Vendéen	0.42 (0.17 - 0.86)
Border Collie*	0.42 (0.28 - 0.59)
English Springer Spaniel*	0.38 (0.23 - 0.60)
Siberian Husky	0.36 (0.13 - 0.79)
Collie Rough	0.36 (0.17 - 0.65)
Drever*	0.32 (0.17 - 0.54)
Cavalier King Charles Spaniel*	0.31 (0.19 - 0.46)
Welsh Springer Spaniel	0.29 (0.11 - 0.64)
Bearded Collie	0.28 (0.08 - 0.73)
Danish-Swedish Farmdog*	0.28 (0.15 - 0.49)
Rhodesian Ridgeback*	0.25 (0.09 - 0.55)
Shih Tzu*	0.25 (0.09 - 0.54)
Schnauzers Miniature*	0.24 (0.14 - 0.40)
German Hunting Terrier	0.19 (0.02 - 0.69)
Chihuahua*	0.18 (0.11 - 0.29)
Pug*	0.18 (0.07 - 0.40)
Dalmatian*	0.17 (0.03 - 0.49)
Münsterländer Small	0.16 (0.02 - 0.59)
Finnish Spitz	0.15 (0.02 - 0.54)
Chinese Crested*	0.13 (0.04 - 0.29)
Norwegian Elkhound Grey*	0.12 (0.04 - 0.28)
Finnish Lapphund*	0.11 (0.02 - 0.33)
Tervueren	0.10 (0.00 - 0.56)
Irish Red Setter	0.10 (0.00 - 0.56)
Dachshund Miniature	0.09 (0.00 - 0.52)
Dachshund Standard*	0.07 (0.03 - 0.13)
Miniature Pinscher*	0.05 (0.00 - 0.28)
Basenji	0 (0 - 0.62)
Basset Hound	0 (0 - 0.89)
Briard	0 (0 - 0.66)
Collie Smooth	0 (0 - 0.82)
German Spitz Medium	0 (0 - 0.62)
Greyhound	0 (0 - 0.57)
Italian Greyhound	0 (0 - 0.63)
Norrbottenspitz	0 (0 - 0.47)

*Increased or decreased RR (relative to the rest of the population with the breed excluded) after Bonferroni correction

CI confidence interval.

Supplementary table 4. The breeds with increased or decreased RR of death/euthanasia due to cruciate ligament rupture (relative to the rest of the population with the breed excluded) in a cohort of dogs insured in Agria Pet Insurance in Sweden during 2011-2016. All RRs were significantly different from 1 ($p < 0.05$)

Breed	Relative risk (95% CI)
Neapolitan Mastiff	66.8 (8.07 - 242.9)
Perro de Presa Mallorquin/ca	44.2 (1.12 - 247.6)
Dogue de Bordeaux*	30.4 (16.5 - 51.5)
Tibetan Mastiff	15.9 (1.92 - 57.9)
Dogo Canario	15.1 (1.83 - 54.9)
American Bulldog	14.6 (3.00 - 43.0)
Chow Chow	14.6 (3.96 - 37.7)
Cane Corso*	12.7 (7.04 - 21.2)
Bullmastiff	9.73 (3.14 - 22.9)
Great Pyrenees	9.48 (2.57 - 24.5)
Newfoundland*	9.10 (3.32 - 20.0)
St. Bernhard	7.25 (1.49 - 21.3)
English Bulldog	7.23 (2.34 - 17.0)
Bolognese	7.10 (2.29 - 16.7)
Rottweiler*	6.56 (4.53 - 9.23)
Boxer*	5.95 (3.30 - 9.93)
Bernese Mountain Dog	5.65 (3.20 - 9.28)
Labrador Retriever*	2.09 (1.49 - 2.88)
Norwegian Elkhound Grey	0.18 (0.00 - 0.99)
Cavalier King Charles Spaniel	0.11 (0.00 - 0.59)
Chihuahua	0.09 (0.00 - 0.50)
Chinese Crested	0 (0 - 0.75)
Dachshund Standard*	0 (0 - 0.26)
English Springer Spaniel	0 (0 - 0.68)

*Increased or decreased RR (relative to the rest of the population with the breed excluded) after Bonferroni correction

CI confidence interval.
